# Identification and validation of neurotrophic factor-related gene signatures in glioblastoma and Parkinson’s disease

**DOI:** 10.3389/fimmu.2023.1090040

**Published:** 2023-02-07

**Authors:** Songyun Zhao, Hao Chi, Qian Yang, Shi Chen, Chenxi Wu, Guichuan Lai, Ke Xu, Ke Su, Honghao Luo, Gaoge Peng, Zhijia Xia, Chao Cheng, Peihua Lu

**Affiliations:** ^1^ Department of Neurosurgery, Wuxi People’s Hospital Affiliated to Nanjing Medical University, Wuxi, Jiangsu, China; ^2^ Clinical Medical College, Southwest Medical University, Luzhou, China; ^3^ Clinical Molecular Medicine Testing Center, The First Affiliated Hospital of Chongqing Medical University, Chongqing, China; ^4^ Department of Oncology, Wuxi People’s Hospital Affiliated to Nanjing Medical University, Wuxi, Jiangsu, China; ^5^ Department of Epidemiology and Health Statistics, School of Public Health, Chongqing Medical University, Chongqing, China; ^6^ Department of Oncology, Chongqing General Hospital, Chongqing, China; ^7^ Department of Radiology, Xichong People’s Hospital, Nanchong, China; ^8^ Department of General, Visceral, and Transplant Surgery, Ludwig-Maximilians-University Munich, Munich, Germany; ^9^ Department of Clinical Research Center, Wuxi People’s Hospital of Nanjing Medical University, Wuxi, Jiangsu, China

**Keywords:** PD, GBM, NFRG, immune cell infiltration, machine learning

## Abstract

**Background:**

Glioblastoma multiforme (GBM) is the most common cancer of the central nervous system, while Parkinson’s disease (PD) is a degenerative neurological condition frequently affecting the elderly. Neurotrophic factors are key factors associated with the progression of degenerative neuropathies and gliomas.

**Methods:**

The 2601 neurotrophic factor-related genes (NFRGs) available in the Genecards portal were analyzed and 12 NFRGs with potential roles in the pathogenesis of Parkinson’s disease and the prognosis of GBM were identified. LASSO regression and random forest algorithms were then used to screen the key NFRGs. The correlation of the key NFRGs with immune pathways was verified using GSEA (Gene Set Enrichment Analysis). A prognostic risk scoring system was constructed using LASSO (Least absolute shrinkage and selection operator) and multivariate Cox risk regression based on the expression of the 12 NFRGs in the GBM cohort from The Cancer Genome Atlas (TCGA) database. We also investigated differences in clinical characteristics, mutational landscape, immune cell infiltration, and predicted efficacy of immunotherapy between risk groups. Finally, the accuracy of the model genes was validated using multi-omics mutation analysis, single-cell sequencing, QT-PCR, and HPA.

**Results:**

We found that 4 NFRGs were more reliable for the diagnosis of Parkinson’s disease through the use of machine learning techniques. These results were validated using two external cohorts. We also identified 7 NFRGs that were highly associated with the prognosis and diagnosis of GBM. Patients in the low-risk group had a greater overall survival (OS) than those in the high-risk group. The nomogram generated based on clinical characteristics and risk scores showed strong prognostic prediction ability. The NFRG signature was an independent prognostic predictor for GBM. The low-risk group was more likely to benefit from immunotherapy based on the degree of immune cell infiltration, expression of immune checkpoints (ICs), and predicted response to immunotherapy. In the end, 2 NFRGs (EN1 and LOXL1) were identified as crucial for the development of Parkinson’s disease and the outcome of GBM.

**Conclusions:**

Our study revealed that 4 NFRGs are involved in the progression of PD. The 7-NFRGs risk score model can predict the prognosis of GBM patients and help clinicians to classify the GBM patients into high and low risk groups. EN1, and LOXL1 can be used as therapeutic targets for personalized immunotherapy for patients with PD and GBM.

## Introduction

Glioblastoma, also known as glioblastoma multiforme (GBM), is classified as a grade IV glioma by the World Health Organization and is the most common primary brain tumor, and the most aggressive form of malignancy (1). Despite the significant advances in molecular understanding of GBM pathogenesis, such as the IDH mutation status (2), the median patient survival time is just 14–16 months, and the 5-year survival rate is only 6.8% (3). The prognosis for GBM patients is still poor, despite rigorous treatment strategies such as surgical resection, radiation therapy, and chemotherapy. Most of the molecular targeted therapies and immunotherapies are in clinical trials, there is need for the development of more effective treatment strategies for GBM (4–6).

Parkinson’s disease (PD) is the second most common neurological disorder after Alzheimer’s disease, which affects roughly 1.2% of individuals over 65 (7, 8). The primary symptom of Parkinson’s disease is loss of motor coordination brought on by the degradation of dopamine neurons in the substantia nigra (SN), which is followed by striatal dopaminergic depletion and the development of Lewy bodies (PD) (9, 10). Factors such as oxidative stress, aging, genetics, and environmental factors may all have a role in the degenerative loss of dopaminergic neurons in Parkinson’s disease (11).

Cancer is characterized by unrestrained cell growth and resistance to cell death, which is in contrast to the excessive neuronal cell death observed in PD (12). In depth analysis of the pathogenesis of the two diseases suggests that patients with neurodegenerative diseases such as PD are less likely to develop cancer (13). Reports from epidemiological studies also point to a decreased risk of main nerve center (CNS) tumors in Parkinson’s disease patients (14, 15). At the genomic level, genes such as the p53 tumor suppressor gene and the epidermal growth factor receptor EGFR that are downregulated in PD are often upregulated in tumors (16, 17). Therefore, there is need for better understanding of potential pathological mechanisms and genetic targets of PD and GBM that will help identify possible shared drug targets to treat both diseases.

Nerve growth factor (NGF), brain-derived growth factor (BDNF), and other proteins that make up the neurotrophic factors family are crucial for the growth, survival, and apoptosis of neurons (18). Neurotrophic factors regulate cell development and apoptosis by interacting with extracellular receptors and transmitting signals about neuronal cell survival and apoptosis to the cell interior (19). Several studies have shown that BNDF expression is reduced in patients with several neurodegenerative diseases, including Parkinson’s disease, and that reduced BDNF levels are an important cause of cognitive impairment in these patients (20). Additionally, numerous studies carried out on animal models have demonstrated that raising plasma BDNF levels may enhance cognition (21–23). On the other hand, neuronal proliferation in the tumor microenvironment is essential for the development of cancer, and neurotrophic factors are essential for the communication between tumor cells and nerves (24). Elevated plasma levels of BDNF have been found in several types of cancer and play an important role in tumor proliferation, survival, migration, and invasion (25). Neurotrophic growth factors generated by cancer cells can also stimulate the formation of neurons in solid tumors, while the release of neurotransmitters from nerve endings stimulates tumor growth and enhances tumor angiogenesis (26, 27). NGF regulates glioma growth and induces cell differentiation through the involvement of the Promyosin receptor kinase A (TrkA) receptor (28). Astrocytes mediate paracrine secretion through glial cell-derived neurotrophic factor (GDNF) and RET (Rearranged during Transfection) signaling to regulate glioma cell invasion. The knockdown of GDNF or its receptor in glioma cells significantly reduces tumor progression *in vitro* (29, 30).

The recent advancements in molecular biology and microarray sequencing technologies has led to the identification of new biomarkers with prognostic and diagnostic potential for various neurodegenerative diseases and neuro-oncology (31, 32). Although several studies have investigated the role of neurotrophic factors in various cancers, neurodegenerative diseases, and cerebrovascular lesions, there is still a gap in identifying neurotrophic factor-related genes with diagnostic potential for PD and exploring immunotherapeutic targets affecting the prognosis of GBM. In this study, GEO and TCGA datasets were used analyze the relationship between differences in expression of NFRGs and the diagnosis of PD and the prognosis of GBM. We then analyzed the potential of two NFRGs—EN1 and LOXL1—as therapeutic targets common to PD and GBM. We also developed a prognostic model for GBM based on NFRGs to showcase the value of NFRGs in predicting the prognosis of GBM patients, enhancing the diagnosis of PD patients, and exploring more efficient personalized therapeutic regimens through a thorough analysis of genomic data and clinically relevant data.

## Materials and methods

### Source of raw data

Three PD datasets, GSE7621, GSE20163, and GSE49036, were downloaded from the NCBI Gene Expression Omnibus (GEO; https://www.ncbi.nlm.nih.gov/geo/). The GSE7621 and GSE49036 datasets were generated using the GPL570 (HG-U133 Plus 2) Affymetrix Human Genome U133 Plus 2.0 array, while the GSE2016 dataset was generated using GPL96 [HG-U133A] Affymetrix Human Genome U133A array. GSE7621 dataset consisted of 16 brain nigrostriatal samples from Parkinson’s disease patients and 9 normal nigrostriatal samples from controls. GSE20163 dataset, which served as an external validation cohort, consisted of 8 PD brain substantia nigra samples and 9 control samples. GSE49036 was used as a validation cohort for clinical staging and included brain substantia nigra samples from 8 Braak stage 0, 5 Braak stages 1-2, 7 Braak stages 3-4 and 8 Braak stages 5-6 patients.

**Figure 1:**
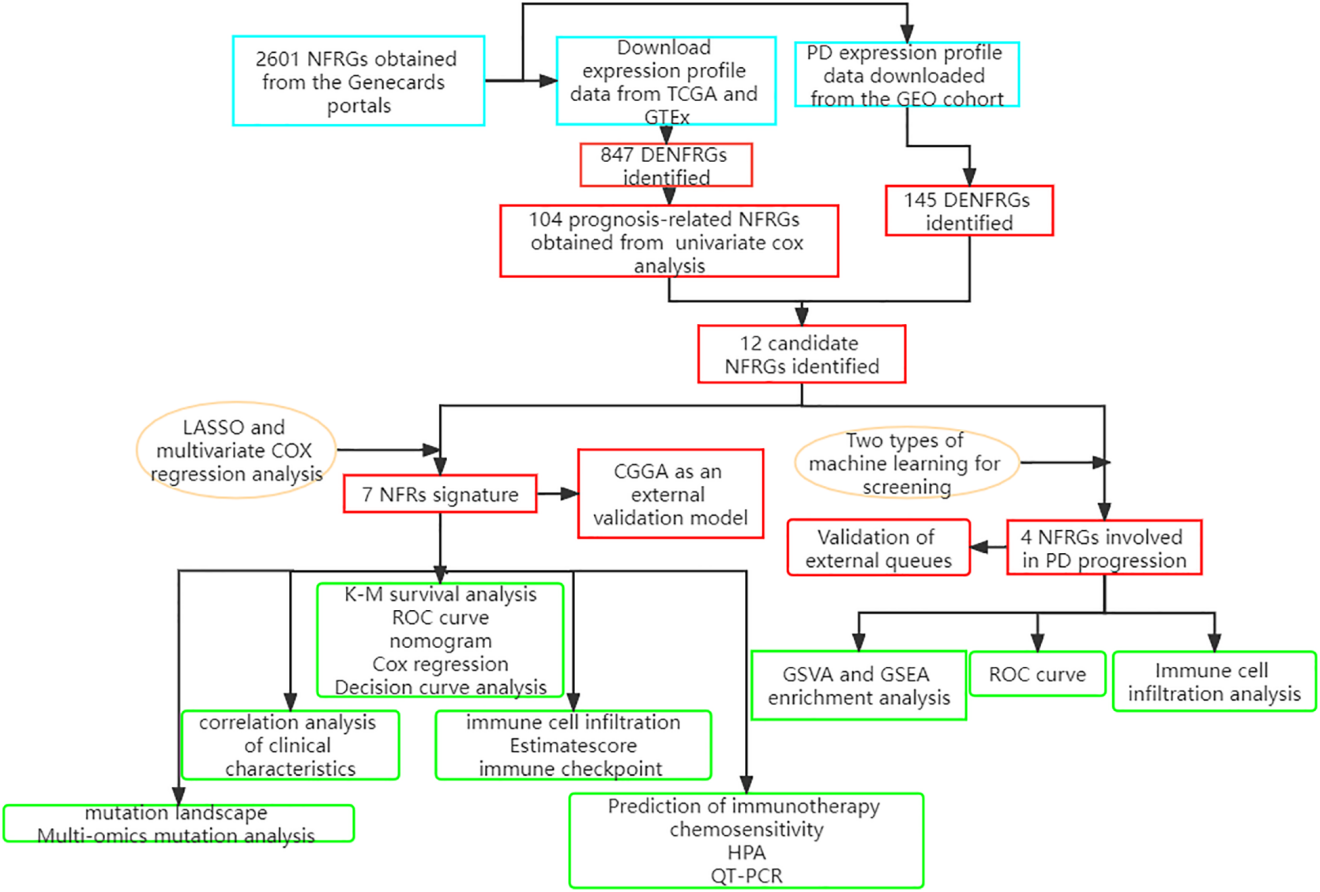
A detailed flow chart showing the NFRGs in GBM and PD.

RNAseq data, mutation data, and clinicopathological characteristics of TCGA-GBM, consisting of 169 glioma samples, were retrieved from the UCSC Xena website (https://xena.ucsc.edu/) Gene expression data for 249 glioma patients were retrieved from the China Glioma Genome Atlas (CGGA) data portal (http://www.cgga.org.cn) and were used to generate a validation model. All expression data were retrieved in TPM format. Batch correction and integration of the two sets of gene expression data were carried out using the “limma” and “sva” (33) packages in R. The detailed flow chart is shown in **Figure 1**.

2601 neurotrophic factor-related genes were downloaded from the GeneCard database (https://www.genecards.org/) (34). Differential gene expression analysis was performed on the TCGA cohort using the “limma” package in R, with | log2FC | > 1.0 and FDR (false discovery rate) < 0.05 as the thresholds. The cutoff p-value of the differentially expressed NFRGs (DENFRGs) for the GEO cohort was set to 0.05, which satisfied the condition of |log2FC|>0.5 The “affy” package in R was used to perform background calibration, normalization, and log2 conversion on all GEO raw data sets (35, 36). The expression values of multiple probes that matched the same gene were averaged. Protein interactions and gene enrichment analysis was carried out using the differentially expressed genes identified from the GEO cohort. The hub genes in the network were screened and visualized using “Cytoscape” software following PPI (Protein-Protein Interaction Networks) analysis on the String online platform.

### Characteristic genes in Parkinson’s disease

To identify signature genes, we used two machine learning methods: LASSO regression analysis and random forest. LASSO is utilized as a dimensionality reduction approach to perform variable screening and complexity adjustment when fitting a generalized linear model. The LASSO analysis was carried out with a penalty parameter and a 10-fold cross-verification using the glmnet program (37). RF (Random Forest) is a combination of classifiers with a tree-like structure, and a minimum error regression tree was built to select key variables using the software package “randomForest”. After tenfold cross-validation, the eight genes with the highest relative importance were used to measure predictive performance.

### Functional enrichment and gene set enrichment analysis

The “clusterProfiler” package of R software was used to carry out the functional enrichment analysis, which included KEGG and GO analysis (38). We adjusted the P values using the Benjamini - Hochberg (BH) technique. A computational technique was used in the gene set enrichment analysis to identify genes that exhibited statistically significant and consistent changes between two biological states. 10,000 permutation tests were used to determine the most important and pertinent signaling pathways. Genes with a corrected P-value and false discovery rate (FDR) below 0.05 were considered to be significant. Statistical analysis and ridge mapping were carried out using the “clusterPro” package in R, which is a non-parametric unsupervised analytic method that is widely employed to evaluate gene set enrichment outcomes in microarrays and transcriptomes. It is primarily used to determine if certain metabolic pathways are enriched across samples by transforming the expression matrix of genes across samples into the expression matrix of gene sets (39). From MSigDB, 50 reference gene sets for hallmark genes were chosen. Gene set variation analysis (GSVA) using the ‘GSVA’ package in R was performed to provide insight into the heterogeneity of biological processes between different clusters.

### Development and validation of prognostic features in GBM

Batch effects between TCGA and CGGA data were removed by creating precise models using the “sva” package in R. Selected NFRGs underwent Minimum Absolute Shrinkage and Selection Operator (LASSO) regression analysis, with the “glmnet” package in R being used to minimize the number of genes in the final risk model. Models were then built using multivariate Cox regression analysis using the following equation: risk score = (Expi), where Expi was the expression value for each NFRG and was the matching regression coefficient (40, 41). The median risk score was used to split all patients into high- and low-risk groups. The “survminer” and “ggrisk” packages in R were used to create survival curves and risk maps to display the disparities in survival and status of each patient. A separate external cohort, the CGGA cohort, was also employed to evaluate the effectiveness of the prognostic model.

A nomogram was created using risk score and clinicopathological features. Calibration charts were internally validated to ensure accuracy of the models. Decision curve analysis (DCA) was carried out using “ggDCA” package in R to evaluate the net clinical benefit of the models (42). We also plotted subject operating characteristic curves using the “timeROC” package in R to evaluate how well risk scores performed in predicting 1-year, 3-year, and 5-year OS in LGG patients (43).

### Prognostic characteristics of the tumor immune microenvironment and mutation landscape

The relative enrichment scores of tumor-infiltrating immune cells (TIICs) were calculated using the R script ssGSEA (single-sample genomic enrichment analysis). We utilized CIBERSORT to calculate and compare the proportion of immune cell types between the low- and high-risk categories, with the sum of all anticipated immune cell type scores in each sample being equal to 1 (44). The TICCs data was downloaded from TIMER 2.0 (http://timer.cistrome.org). The results from TIMER, CIBERSORT, amounts, MCP-counter, xCELL, and EPIC algorithms were also compared between the two groups. The “oncoplot” function in the “maftools” package of the R software was used to create two waterfall plots to compare the specific mutation characteristics between the high- and low-risk groups.

### Gene set cancer analysis database

The tumor genomic analysis platform GSCALite (http://bioinfo.life.hust.edu/web/GSCALite/) integrates genomic data for 33 tumor types from the TCGA library, GDSC (Genomics of Drug Sensitivity in Cancer), CTRP (The Cancer Therapeutics Response Portal) medication response data, and normal tissue data from GTEX (Genotype-Tissue Expression) for comprehensive genomic analysis (45).

### Immunotherapeutic response prediction and drug sensitivity assessment

The Immunological Cell Abundance Identifier (ImmuCellAI) is a computer program launched in 2020 to predict immunological checkpoint reactions based on the abundance of TICCs, particularly certain T cell subpopulations. Comprehensive immunogenomic analysis findings are provided by the Cancer Immunome Atlas (TCIA) online software. Using a scale from 0 to 10, the Immunophenotype Score (IPS) quantifies the immunogenicity of tumors (46). IPS can be used to predict response to immune checkpoint inhibitors. The “prophytic” package in R was used to compute the half-maximal inhibitory concentration (IC50) of samples from the high and low risk score groups in order to test the ability of the risk score to predict sensitivity of samples to chemotherapy and molecular medicines. Zaoqu Liu et al. from the First Affiliated Hospital of Zhengzhou University developed THE BEST website (http://rookieutopia.com/). The database contains sequencing data from a variety of tumors after treatment with immune checkpoint inhibitors

### Tumor Immune Single Cell Hub database

Tumor Immune Single-Cell Hub (TISCH; http://tisch.comp-genomics.org) is an extensive single-cell RNA-seq database dedicated to TME. It enables comprehensive analysis of TME heterogeneity across different datasets and cell types.

### QT-PCR and immunohistochemistry

Human astrocytes (HA), U87 and A172 glioma cells, obtained from the Center for Experimental Medicine, Southwestern Medical University. All cells were grown in 10% fetal bovine serum-supplemented DMEM. Cells were incubated at 5% CO2 and 37°C. TRIzol reagent was used to isolate RNA, while PrimeScriptTM RT kit was used to perform reverse transcription. Quantitative PCR was carried out using Takara’s SYBR Green PCR Master Mix on the StepOnePlus system. Ploidy changes at the gene level were determined using the 2-ΔΔCT method, with GAPDH as the normalization gene. The primer sequences involved in this study are as follows.

EN1:FORWARD : GAAGAACGAGAAGGAGGACAAGCG,REVERSE: CGTGGTGGTGGAGTGGTTGTAC.

LOXL1:FORWARD : GAAGAACCAGGGCACAGCAGAC,REVERSE ATGTCCGCATTGTAGGTGTCATAGC.

GAPDH:FORWARD : ATGGGGAAGGTGAAGGTCG,REVERSE : GGGGGTCATTGATGGCAACAATA. Each PCR reaction was performed in triplicate.

Transcriptomics and proteomics methods were used to study protein expression at the RNA and protein levels in human tissues and organs, using data found in Human Protein Atlas (HPA, https://www.proteinatlas.org/).

### Statistical analysis

All analyses were conducted using R version 4.1.1, 64-bit6. Prognosis and patient survival in various subgroups were compared using Kaplan-Meier survival analysis and the log-rank test. The nonparametric Wilcoxon rank sum test was used to compare continuous variables between the two groups, while Kruskal-Wallis test was employed for comparisons among more than two groups. Univariate and multivariate Cox regression (R package “survival”) analyses were used to identify clinical traits with prognostic potential in the high- and low-risk groups. Spearman correlation analysis was used to assess correlation coefficients. P < 0.05 was regarded as statistically significant in all statistical investigations. The ROC curves, the nomogram model and the Concordance Index were generated using the “survivalROC”, “rms” and the “pec” (C-index) packages in R, respectively. The changes in gene expression between the two isoforms were determined using principal component analysis (PCA).

## Results

### Identification of neurotrophic factor-related genes associated with Parkinson’s disease and GBM

**Figure 2:**
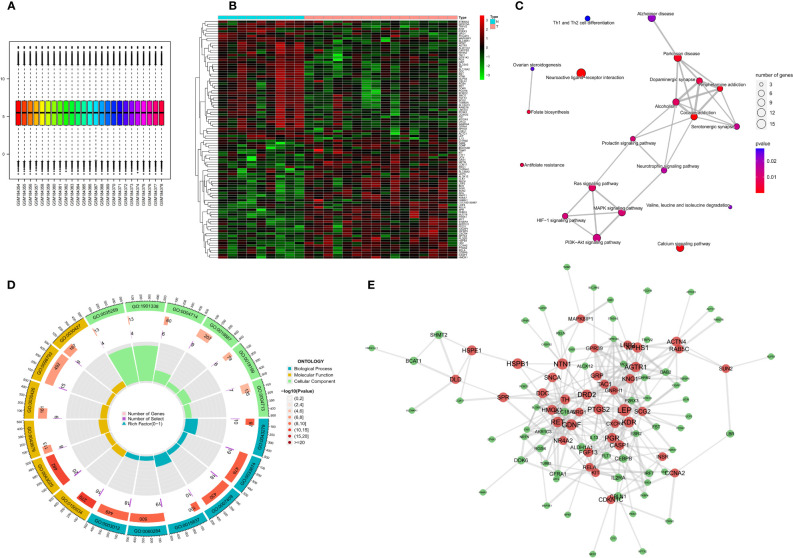
Expression of differential NFRGs in Parkinson’s disease, enrichment analysis, and construction of protein interaction network. **(A)** Box line plot of the GSE7621 dataset samples corrected for batch-to-batch differences after removal. **(B)** Heat map showing the expression of all DEGs in Parkinson’s samples. **(C)** Network diagram of KEGG enrichment analysis. **(D)** Circle diagram of GO enrichment analysis. **(E)** Interaction plots of proteins. Red represents hub genes.

The limma package was used for background correction and normalization of expression data from the GSE7621 dataset. Batch effects were removed using the sva package, and the box plot corresponding to the processing results is shown in **Figure 2A**. The expression of each of the 145 differentially expressed genes (DEGs) in the GEO cohort is shown in **Figure 2B**. GO analysis revealed that the DEGs were enriched in “positive control of kinase activity” in the biological process (BP) category, “neuronal cell body” in the cellular component (CC) category and the “nuclear glucocorticoid” in the Molecular Function (MF) category (**Figure 2D**). KEGG pathway analysis showed that the DEGs were more closely related to “Neuroactive ligand-receptor interaction” (**Figure 2C**). We also constructed PPI networks to investigate the interaction of the proteins encoded by the DEGs based on betweenness centrality, with the central genes in the network being marked in red. (**Figure 2E**).

**Figure 3:**
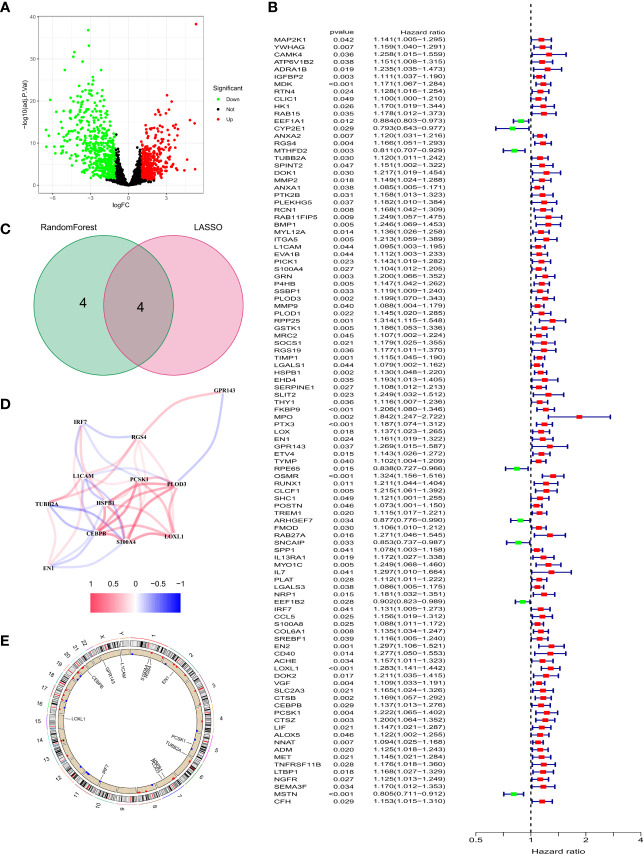
Identification of prognosis-related NFRGs in GBM patients in the TCGA cohort. **(A)** Volcano plot of DEGs. **(B)** Forest plot of univariate cox analysis. **(C)** The intersection of DEGs and univariate cox results for the GEO cohort. **(D)** Correlation analysis of 12 NFRGs. **(E)** Chromosomal localization of 12 NFRGs.

We identified 847 DEGs from differential gene expression analysis of NFRGs between tumor and normal tissues of the TCGA-GBM cohort. Out of the total DEGs, 489 genes were down-regulated whereas 358 genes were up-regulated in tumor tissue (**Figure 3A**). Univariate Cox regression analysis identified 104 differentially expressed NFRGs with prognostic potential for GBM in the TCGA cohort (**Figure 3B**). We then determined the NFRGs that overlapped from the univariate cox analysis of the TCGA cohort and the differentially expressed NFRGs obtained from the GEO cohort. A total of 12 NFRGs overlapped between the two cohorts indicating that they were associated with the occurrence of Parkinson’s disease and prognosis of GBM (**Figure 3C**). **Figure 3D** shows the correlation of the expression of these 12 NFRGs in the TCGA cohort, while **Figure 3E** shows the localization of these 12 NFRGs on chromosomes, with EN1 being localized on chromosome 2.

### Selection of Parkinson’s disease signature genes using LASSO regression and random forest algorithm

**Figure 4:**
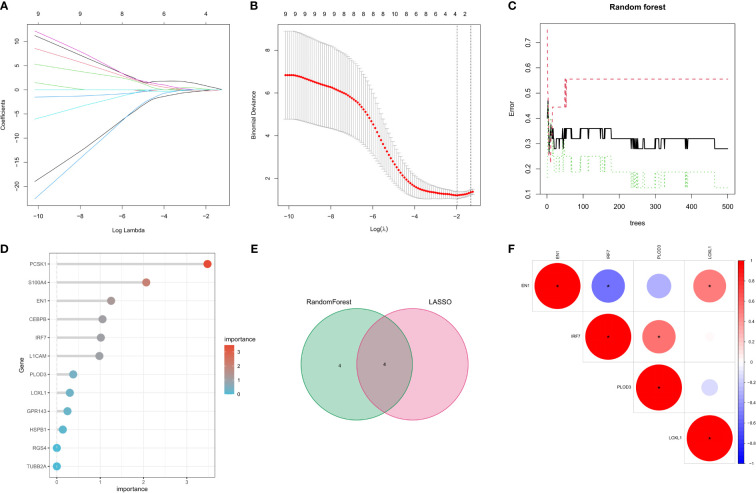
Selection of Parkinson’s disease-related hallmark genes among NFRGs. **(A)** Ten cross-validations of the LASSO model’s improved parameter selection. Each curve represents on gene. **(B)** Construction of linear models (Lasso) and visualization by coefficients. **(C)** The best lambda is where vertical dashed lines are drawn. The error rate for random forests with the number of classification trees. **(D)** Importance ranking of all selected genes. **(E)** lasso regression analysis and random forest for the intersection of genes. **(F) **Spearman correlation analysis of the four NFRGs. *p < 0.05, **p < 0.01, ***p < 0.001.

Two machine learning algorithms were used to identify key genes among the 12 NRFGs. The best lambda for the LASSO algorithm was 0.138 after ten cross-validations. Due to higher accuracy in comparisons, we used the minimum criterion for the LASSO classifier, and identified 4 key genes, including IRF7, EN1, PLOD3, and LOXL1 (**Figures 4A, B**). The influence of the number of decision trees is shown in **Figure 4C**. The x-axis shows the number of decision trees, while the y-axis shows the mistake rate. The top 8 key genes with relative relevance scores identified using the random forest technique were PCSK1, S100A4, EN1, CEBPB, IRF7, L1CAM, PLOD3, and LOXL1 (**Figure 4D**). Four key genes, IRF7, EN1, PLOD3, and LOXL1, overlapped from results of lasso regression and random forest algorithm analysis (**Figure 4E**). **Figure 4F** shows the correlation of these four feature genes in the GEO cohort.

### Diagnostic efficacy and enrichment analysis of characteristic genes

**Figure 5:**
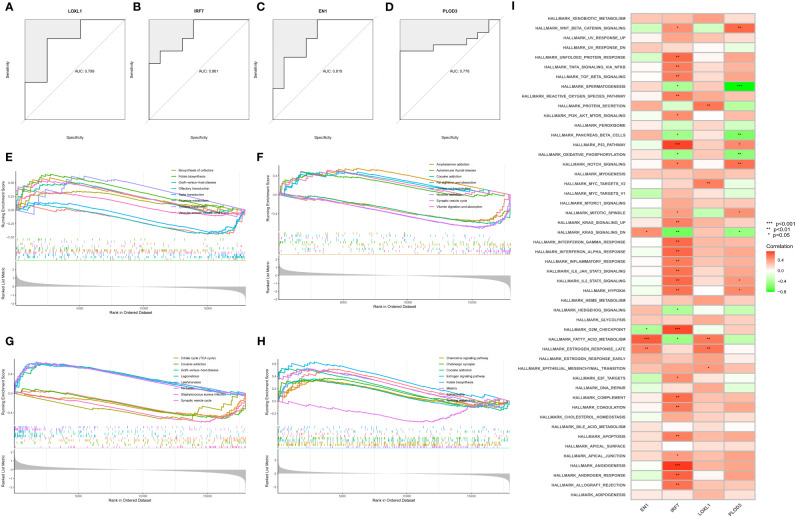
Construction, diagnostic efficacy, and enrichment analysis of histograms of characteristic NFRGs. **(A–D)** ROC curves for calculating the signature genes’ diagnostic performance. **(E–H)** The main signaling pathways associated with specific genes identified using GSEA. **(I)** Correlation of signature genes with pathways using GSVA analysis.

We then estimated the diagnostic performance of the four key genes. The AUC values of the ROC curves were 0.799 for LOXL1 (**Figure 5A**), 0.778 for PLOD3 (**Figure 5B**), 0.861 for IRF7 (**Figure 5C**), and 0.819 for EN1 (**Figure 5D**). GSEA was used to evaluate the signaling pathways associated with the signature genes. Our results showed that LOXL1 (**Figure 5E**), PLOD3 (**Figure 5F**), IRF7 (**Figure 5G**), and EN1 (**Figure 5H**) were mainly associated with functions of the nervous system and the transmission of neurotransmitters. For example, EN1 was strongly correlated with spinal cerebellar ataxia and dopaminergic synapse-related pathways. The GSVA results demonstrated the correlation of the four signature genes with the HALLMARK pathway (**Figure 5I**).

### Assessment of the microenvironment in PD

We also quantified the ssGSEA enrichment scores for several immune cell subpopulations, associated PD functions or pathways, and healthy controls. A heat map was used to display the number of TIICs and immunological responses in each sample (**Supplementary Figure 1A**). **Supplementary Figures 1B, C** show heat maps displaying the relationship between TIICs and immune function, with darker red denoting a stronger correlation between the two. The association between the four key NFRGs and immune-related pathways in the ssGSEA data was also demonstrated using a heat map (**Supplementary Figure 1D**). These results indicated that the four NFRGs play a role in the immune microenvironment of PD.

### Internal and external data validation of characteristic genes

**Figure 6:**
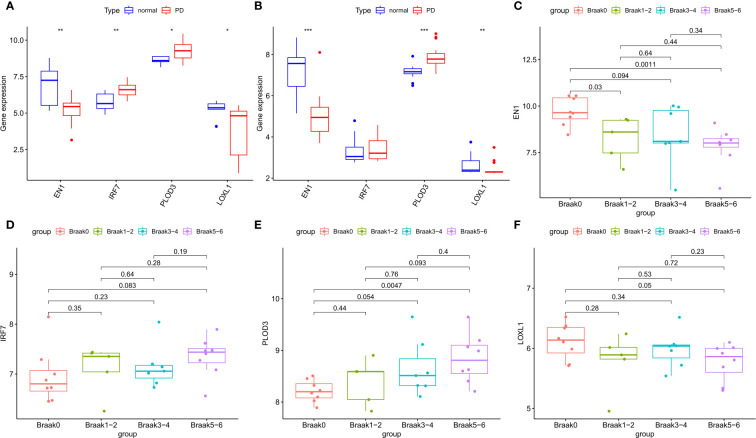
Expression validation of characteristic NFRGs. **(A) **Characterization of NRGs expression in the internal validation cohort (GSE7621). **(B) **Characterization of NRGs expression in the external validation cohort (GSE20163). **(C–F)** Expression of characteristic NRGs in different stages of PD (GSE49036).

In the GSE7621 internal validation cohort, the expression of EN1 and LOXL1 was lower, while the expression of IRF7 and PLOD3 was higher in PD tissues than in normal controls (**Figure 6A**). The expression of the four signature genes in the GSE20163 external validation cohort was similar to that in the internal validation cohort, except for IRF7 (**Figure 6B**). The difference in results may be due to the small sample size. In the GSE49036 dataset consisting of patients with Parkinson’s disease at different Braak stages, there was significant difference in the expression of EN1, PLOD3 and LOXL1 in the different Braak stages 0 to 6 of Parkinson’s disease. However, there was no significant difference in expression of IRF7 among the different stages (**Figures 6C–F**). These results suggest that these NFRGs play a role in the pathogenesis and progression of Parkinson’s disease.

### Construction and validation of predictive models for NFRGs in GBM

**Figure 7:**
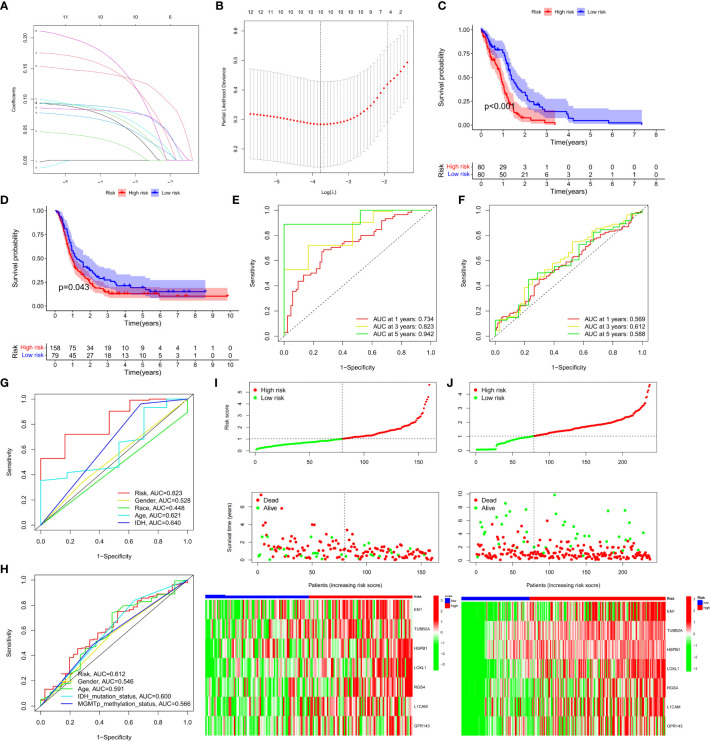
Development and validation of prognostic models for GBM patients. **(A)** The 10-fold cross-validation LASSO analysis found seven prognostic genes. Each curve represents one gene. **(B)** Plots illustrating the coefficient profiles for seven prognostic NRGs. The best lambda is where vertical dashed lines are drawn. **(C, D)** Survival curves showing the risk stratification ability of TCGA and CGGA cohorts. **(E, F)** AUC values for TCGA and CGGA cohort risk groupings at 1, 3, and 5 years. **(G, H)** AUC values for 3-year clinical characteristics and risk groups for the TCGA and CGGA cohorts. **(I, J)** The risk plots of survival status of each sample in the TCGA and CGGA cohorts. Heat map showing the expression of each gene’.

A risk-scoring model was developed based on the 12 NRFGs obtained in **Figure 3** to identify potential prognostic biomarkers for GBM. The NFRGs with prognostic potential were subjected to LASSO regression analysis to reduce the number of genes in the final risk model. Ten NFRGs were identified from this step (**Figures 7A, B**). Multivariate Cox analysis identified 7 NFRGs, including EN1, TUBB2A, HSPB1, LOXL1, RGS4, L1CAM, and GPR143 as independent prognostic factors. Risk scores were calculated using the following formula: risk score = expression level of EN1* 0.17 + expression level of TUBB2A* 0.09 + expression level of HSPB1*0.14+ expression level of LOXL1*0.19 + expression level of RGS4*0.09 + expression level of L1CAM*0.08 + expression level of GPR143*0.20.

Patients in the TCGA cohort were classified into high-risk and low-risk groups based on the median risk score. Survival curves revealed that patients in the high-risk group had lower overall survival (OS) compared to the low-risk group in the TCGA and CGGA cohorts (**Figures 7C, D**, P<0.05). Furthermore, the risk score was effective at predicting OS in the TCGA cohort. (AUCs for 1-, 3-, and 5-year OS were 0.734, 0.823, and 0.942, respectively; **Figure 7E**). However, since GBM patients have dismal prognosis, the AUC values in the CGGA sample were not favorable (**Figure 7F**). In both the TCGA and CGGA cohorts, the area under the curve (AUC) for the risk score over three years was greater than the AUC values for the other clinicopathological features (**Figures 7G, H**). Risk maps were used to display survival results from the TCGA and the CGGA cohorts, while heat maps were used to display variations in the expression of the seven NFRGs across the various risk groups (**Figures 7I, J**).

PCA and t-SNE analyses were then performed using the NFRG classification in the expression profiles of the seven models. In the TCGA cohort (**Supplementary Figures 2A, B**) and the CGGA cohort (**Supplementary Figures 2C, D**), our signatures yielded results indicating a different distribution between the high-risk and low-risk groups. These findings imply that prognostic model can distinguish between high and low risk groups.

### Establishment of a prognostic nomogram and clinical features

**Figure 8:**
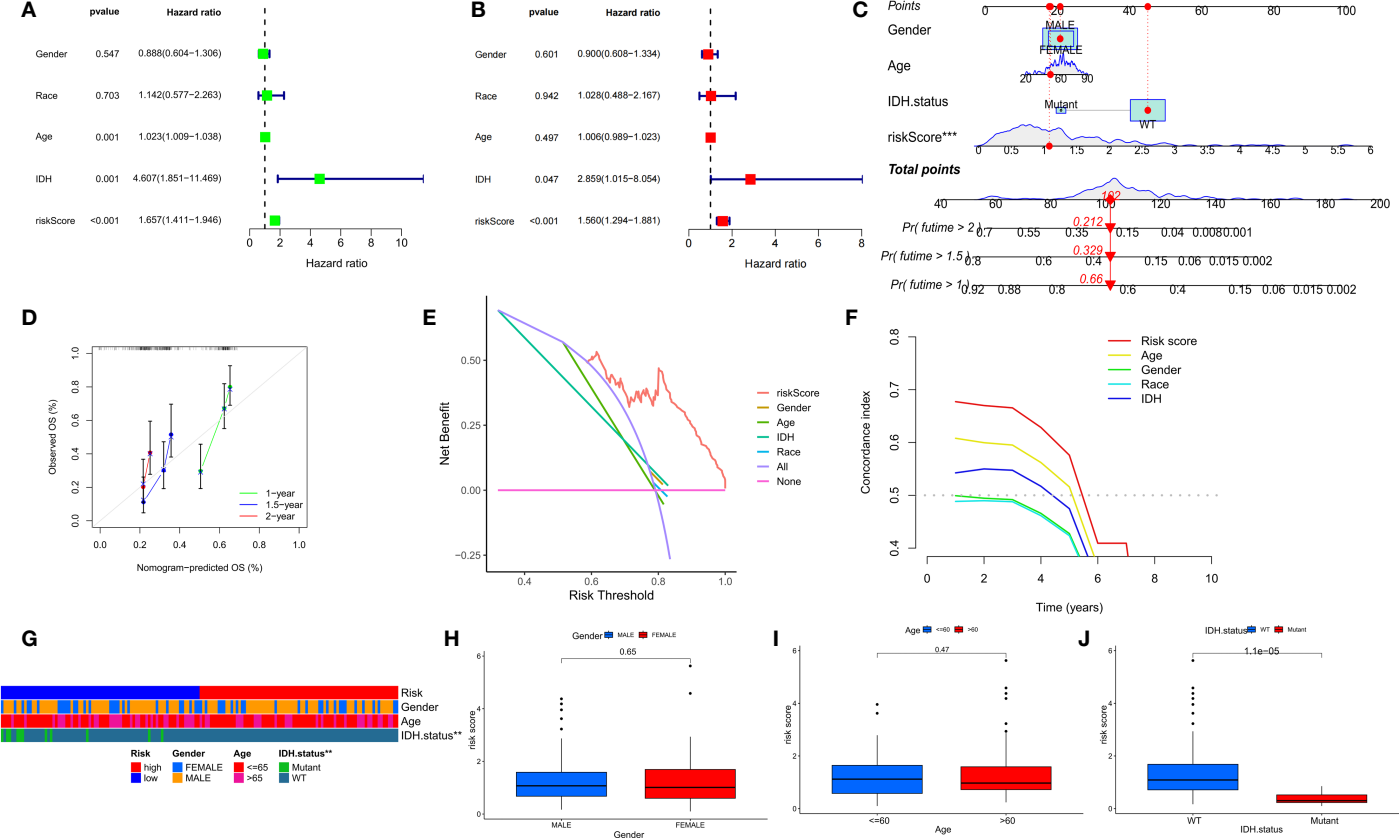
Prognostic value of risk scores and clinical characteristics in GBM patients. **(A)** Univariate and **(B)** multivariate COX analysis for evaluating the prognostic signature and clinical features (including age, race, gender, and IDH state). **(C)** Nomogram of risk groupings and clinical characteristics for predicting survival at 1, 1.5, and 2 years. **(D)** Calibration curves for testing the agreement between actual and predicted outcomes at 1, 1.5, and 2 years. **(E)** DCA curves of risk scores and clinical characteristics for the TCGA cohort at 3 years. **(F)** The concordance index (C-index) for the TCGA cohort. **(G)** Bar charts of clinical characteristics associated with risk grouping determined by chi-square test. **(H–J)** Variations in risk scores among the TCGA cohort’s various clinical characteristic groupings *p < 0.05, **p < 0.01, ***p < 0.001.

Univariate and multivariate Cox analyses showed that risk scores were independent prognostic factors for GBM patients compared to other common clinical characteristics (**Figures 8A, B**). In the CGGA cohort, results of both univariate and multivariate cox analyses showed that risk score was a prognostic factor independent of age, IDH mutation status, or MGMTp_methylation status (**Supplementary Table 1**). To determine the clinical application of the risk models, age, sex, IDH mutation status, and risk score were included in a nomogram used to predict overall survival in patients with GBM based on the TCGA cohort (**Figure 8C**). We found that the risk score had the biggest influence in predicting OS, an indication that prognosis of GBM could be predicted using a risk model based on the seven NFRGs. At 1, 1.5, and 2 years, the calibration curves demonstrated a reasonable agreement between expected and observed values (**Figure 8D**). The three-year DCA curves (**Figure 8E**) and the temporal c-index values (**Figure 8F**) indicated that our model has the highest net benefit and that the risk model constructed based on the 7 NFRGs has more influence in clinical decision-making than the traditional model. The histogram of the chi-square test showed that risk grouping was only associated with whether IDH was mutated (**Figure 8G**). To validate these findings, we evaluated the relationship between risk score and clinical characteristic and found that individuals without IDH mutations were associated with higher risk scores (**Figures 8H–J**).

### NFRGs risk score predicts immune cell infiltration

**Figure 9:**
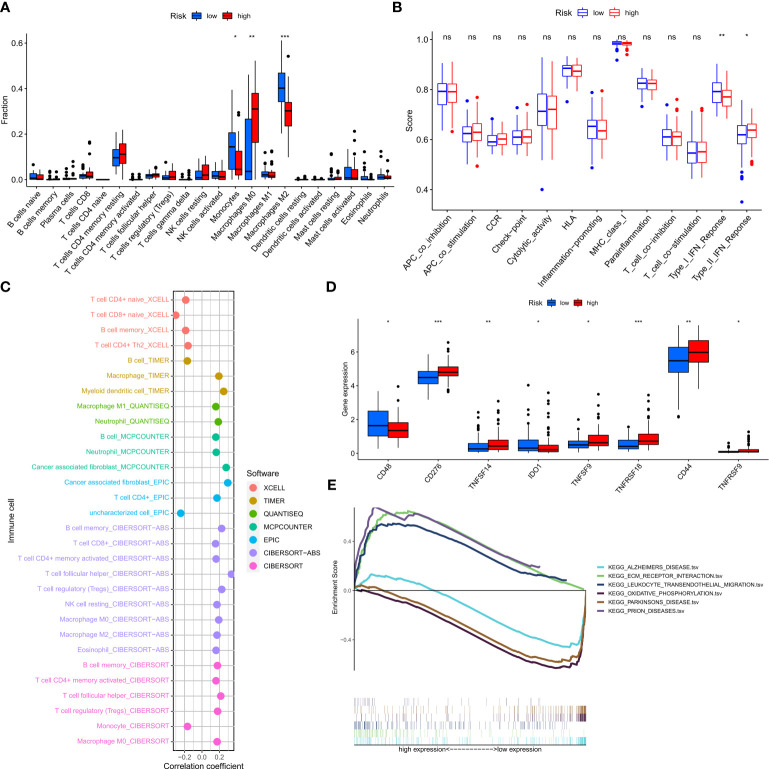
Prediction of the tumor microenvironment and immune cell infiltration by the 7-NFRGs risk score. **(A)** Differences in immune cell infiltration levels between high and low-risk groups. **(B)** Differences in immune function between high and low-risk groups. **(C)** Immune cell bubble map. **(D)** Differences in immune checkpoint between high- and low-risk groups. **(E)** GSEA analysis focusing on the differential enrichment of KEGG pathways. *P < 0.05, **P < 0.01, ***P < 0.001, ns ≥ 0.05.

To determine the relationship between risk scores and immune cells and functions, we measured the enrichment scores of various immune cell subpopulations, associated activities, or pathways using the “cibersort” and “ssGSEA”. The low-risk group was associated with a larger infiltration of monocytes and M2-type macrophages (**Figure 9A**). In addition, the high-risk group showed a higher type 2 interferon response compared to the low-risk group, while the low-risk group had a higher type 1 interferon response (**Figure 9B**). The risk score was associated with the quantity of immune cells in the GBM tumor microenvironment determined by several methods using Spearman correlation analysis (**Figure 9C**). Furthermore, we discovered that a small number of immune checkpoints, namely CD48 and IDO1, were substantially expressed in the low-risk group compared to the high risk group (**Figure 9D**). These results imply that although patients in the high-risk group have a worse prognosis, they may be more responsive to immunotherapy due to their more active immune function. GSEA was used to investigate potential changes in biological function between risk groups based on the various prognoses of patients in the high-risk and low-risk groups. We chose the top 8 enriched signaling pathways based on normalized enrichment scores (NES) and p-values (**Figure 9E**). Surprisingly, lower risk scores were associated with Alzheimer’s disease and Parkinson’s disease, which is in line with the theme of our study.

### Mutation landscape of risk groupings and multi-omics mutation analysis of NFRGs

To determine the molecular mechanisms driving the abnormal expression of these seven NFRGs, we explored the many histological levels, including genomes and copy numbers. Analysis of single nucleotide gene variant (SNV) data revealed that missense mutations in NFRGs were the most frequent variant categorization in the TCGA-GBM cohort, whereas single nucleotide polymorphism was the most common variation type. Among the SNV categories, C>T showed the highest prevalence (**Supplementary Figure 3A**). To summarize the ratio of pure and heterozygous mutations in the sample’s NFRGs, copy number variation was examined (**Supplementary Figure 3B**). We found that 17 GBM patients had mutations, with L1CAM mutations being the most common (**Supplementary Figure 3C**). Additionally, the Spearman’s correlation coefficient analysis of between copy number variations and gene expression showed that L1CAM copy number variations were downregulated in GBM whereas TUBB2A, HSPB1, LOXL1, RGS4, and GPR143 copy number variations were upregulated (**Supplementary Figure 3D**). Heterozygous variants of HSPB1 were present in most samples, and individual analysis showed that LOXL1 and L1CAM were copy number deletions. Whereas the pure-sibling mutation of GPR143 is mainly a copy number reduction (**Supplementary Figures 3E, F**), HSPB1 and L1CAM primarily amplified pure heterozygous mutations, suggesting that abnormal gene expression may be caused by both copy number variation and single nucleotide variation. The relationship between NFRGs expression and the activity of pathways linked to cancer was further examined. The findings demonstrated that NFRGs contributed to the inhibition of hormonal pathways in GBM patients and activation of the EMT, PI-3K-AKT, and TSC-mTOR pathways (**Supplementary Figure 3G**). We further explored the differential expression of NFRGs in the GDSC and Cancer Therapy Response Portal databases, their corresponding drug sensitivity (**Supplementary Figures 3H, I**). This suggested that the expression of the proposed risk profile genes may be exploited to develop agents for sensitizing drugs as well as predict chemotherapeutic drug sensitivity in patients.

In further experiments, we examined the correlation between risk score and tumor mutational load (TMB) (**Supplementary Figure 4B**) as well as differences in TMB among different risk subgroups (**Supplementary Figure 4A**). Results showed that the TMB was higher in the low-risk group. Thus, we generated two waterfall plots to explore the detailed mutational characteristics between high- and low-risk populations. The results indicated that PTEN, TP53, and TTN were the most commonly mutated genes in both risk groups (**Supplementary Figures 4C, D**).

### NRFGs risk score predicts treatment response assessment

**Figure 10:**
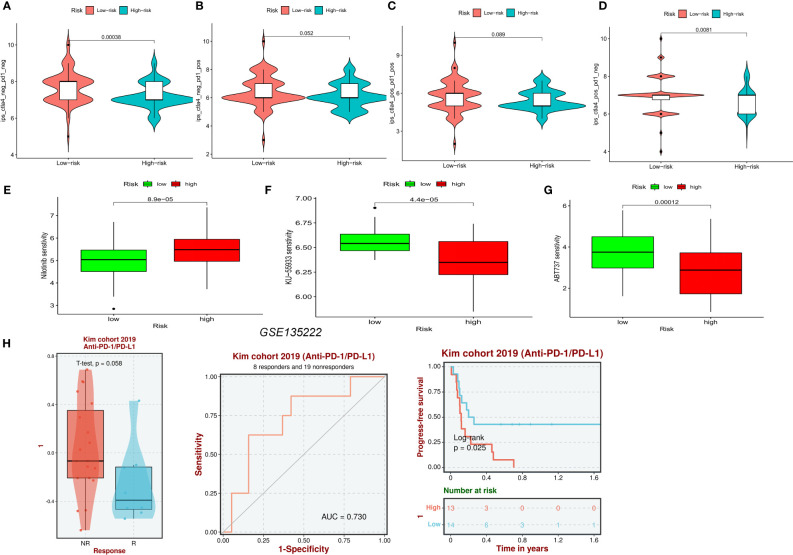
Prediction of pharmaceutical and immunotherapy for various risk groupings. **(A–D)** The comparison of the relative distribution of immunophenoscore (IPS) between high and low-risk groups. **(E–G)** IC50 values for patients in the high- and low-risk groups based on Nilotinib, ABT737, and KU-55933 to assess the sensitivity of chemotherapeutic agents. **(H)** Evaluation of anti-PD-L1 therapy in the GSE135222 cohort by NFRGs.

Analysis of the violin plots designed to demonstrate the link between IPSs and risk groups, showed that high IPSs indicate stronger responses to PD-1 and CTLA-4 blockers (**Figures 10A–D**). Using the “pRRophetic” R package, we explored the potential sensitivity of clinical agents in the high-risk and low-risk groups. Agents available for the treatment of gliomas, such as nilotinib (**Figure 10E**), had a higher IC50 in patients of the high-risk group, whereas ABT737 and KU-55933 had a higher IC50 in patients of low-risk groups (**Figures 10F, G**). To understand the association between risk scores of NFRGs and the benefits of immunotherapy, we investigated a cohort of lung cancer patients treated with PD-1 checkpoint inhibitors (GSE135222) using the BEST database. The ROC curve analysis demonstrated that NFRGs was effective in predicting immunotherapy responsiveness, with low NFRGs expression score correlating with higher degree of immune response to anti-PD-L1 (**Figure 10H**).

### 7 NFRGs in single-cell RNA sequencing

Using the single-cell dataset GSE141982 from the TISCH database, we investigated the expression of 7 NFRGs in the GBM TME. It was observed that the GSE141982 dataset was enriched with several cell types of 16 cell populations and 4 cell subpopulations (**Supplementary Figure 5A**). Most endothelial cells, monocyte macrophages, and CD8+ T cells expressed HSPB1 and TUBB2A. The expression of the other NFRGs, which are primarily found in tumor cell cells, was low (**Supplementary Figures 5B, C**).

### QT-PCR and immunohistochemistry

**Figure 11:**
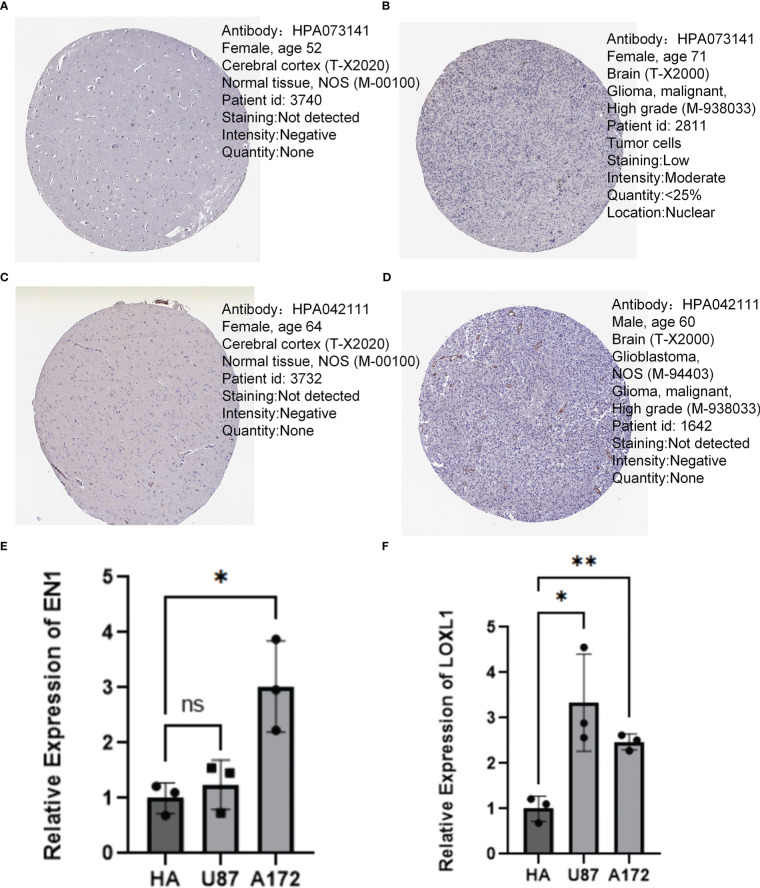
Immunohistochemistry and QT-PCR. **(A, B)** Protein expression levels of EN1 in normal cerebral cortex and GBM. **(C, D)** Protein expression levels of LOXL1 in normal cerebral cortex and GBM. **(E)** RT q-PCR analysis of EN1 expression in various types of glioma cells. **(F)** RT q-PCR analysis of LOXL1 expression in various types of glioma cells. *p < 0.05, **p < 0.01, ***p < 0.001, ns, no significance.

By analyzing the neurotrophic factor-related genes in PD and GBM, we found that EN1 and LOXL1 we important players in both diseases. In the human protein atlas, the protein expression of EN1 (**Figures 11A, B**) and LOXL1 (**Figures 11C, D**) were higher in GBM relative to normal cortical tissue. RT-qPCR results confirmed the higher expression of EN1 and LOXL1 in both GBM cell lines (**Figures 11E, F**).

## Discussion

In clinical practice, the diagnosis of PD is mainly based on neurological examination when patients with PD present with motor symptoms. Currently, the etiology of PD is still not fully understood. For this reason, there is no cure or intervention to delay the progression of the disease, and PD is only treated symptomatically through medication and rehabilitation (47). Moreover, most patients have advanced neurological symptoms at the time of diagnosis (48). Similar to PD, patients with GBM are diagnosed through clinical examination and neuroimaging methods. Therefore, it is imperative to study the underlying pathogenesis of PD and GBM and identified biomarkers for early identification to promote timely treatment of neurological symptoms before they appear.

Although the pathways affected in PD and GBM are highly similar, it has been reported that those that regulate cell proliferation and metabolism play opposite roles in the two diseases. For example, p53 inhibits GBM cell proliferation by blocking cell cycle progression and promoting apoptosis; however, in PD, increased p53 expression upregulates the expression of α-synuclein and its subsequent aggregation in which promotes disease progression (49, 50). The PTEN/PI3K/Akt signaling pathway is down-regulated in PD and up-regulated in GBM (51, 52). An increase in PTEN in PD leads causes inhibition of pro-survival signaling pathways resulting in neuronal cell death. In mouse models, was found that depletion of PTEN attenuated the loss of dopaminergic cells and reduced the symptoms of PD (53). Overexpression of EGFR activated the PTEN/PI3K/Akt signaling pathway in GBM, and mutations in PTEN and phosphorylated Akt have been linked to poor prognosis of GBM patients (52, 54).

Since their discovery, neurotrophic factors have been found to play important roles in many processes such as survival, growth, and differentiation of nerve cells in the peripheral and central nervous systems. It has been found that neurotrophic factors can improve the survival and function of nigrostriatal dopaminergic neurons. They can also promote the survival and synaptic plasticity of mature neurons and protect neurons from damage (55). Neurotrophic factors have also been widely reported in gliomas. For example, GDNF which is released by glioma cells can promote tumor growth, an action that is dependent on the presence of microglia (56). The development of immunotherapy has triggered an increasing number of investigations into the clinical efficacy of targeting immune checkpoints, including early diagnosis, combination therapy, and treatment prediction in patients with various types of tumors. Individual neurotrophic factor family members are now considered to be biomarkers for predicting cancer development and prognosis (57). Overactivation of the immune system concurrently can induce or stimulate the onset of neurodegeneration and cancer, as well as local or systemic inflammatory reactions (58). In GBM, specific cytokines generated by tumor cells suppress the effects of immune response and allow tumor cells to evade the immune system. Elevated cytokine levels induced by cellular stress in PD can result in neuronal cell death (59). In addition to this, BDNF is thought to produce anti-tumor immune responses during the development and differentiation of neurons (60). Currently, few studies have explored the neurotrophic factors in both PD and GBM and identify factors driving the pathogenesis of PD, as well as the associated immune mechanisms.

In this investigation, we first used the analysis of variance and univariate cox to characterize 12 NFRGs influencing the prognosis of GBM and PD development. Subsequently, we applied two machine learning algorithms to analyze the 12 NFRGs and selected four distinctive NFRGs from two external validation cohorts which were thought to potentially affect the development of PD. The lasso regression analysis and multivariate cox analysis were performed on the 12 NFRGs in the TCGA-GBM cohort, resulting in the creation of a 7-NFRGs model. A validation investigation was conducted on the developed NFRGs risk score model and to determine its capacity to accurately predict the prognosis of GBM patients. Based on expression levels of the screened 7-NFRGs, a risk score was generated for each patient, and the patients were classified into high and low-risk groups based on the median risk score. Columnar plots containing clinicopathological variables were created. Calibration curves showed a good correlation between predicted and observed values. In addition, conventional clinical features including age, gender, and IDH mutation status were used to predict the prognosis of GBM. In conclusion, the constructed model had the largest net return, showing that the developed NFRGs risk model is clinically important in decision-making and implementation of individualized anti-tumor treatment.

In this work, we found that seven NFRGs, EN1, LOXL1, TUBB2A, HSPB1, RGS4, L1CAM, and GPR143t, together constitute a stable risk score for GBM. In PD, three NFRGs, EN1, LOXL1, and PLOD3, were identified to influence the disease course of PD patients. EN1 and LOXL1 have the potential to be targets for immunotherapy in GBM and PD patients. The EN1 gene encodes homeobox protein engrailed-1 and its mutations were first discovered to cause abnormal growth and development in Drosophila (61). In humans, EN1 expression affects multiple neuronal cell types and can profoundly regulate central nervous system development (62). Hypermethylation of EN1 has been reported in many cancers, including colorectal cancer, prostate cancer, and glioma, and the degree of methylation correlates with tumor grade and patient prognosis (63–65). In a recent study, Chang et al. found that EN1 can regulate the Hedgehog signaling by modulating Gli1 expression and levels of primary cilia transport-associated protein TULP3. Therefore, be used as a diagnostic and prognostic marker for glioblastoma (66). In addition, it was reported that EN1 participates in the regulation of maturation and survival of midbrain dopaminergic neurons, and polymorphisms in the EN1 gene may be a potential genetic risk factor for sporadic PD (67). In mice models, EN1 and EN2 were found to not influence the survival of dopaminergic neurons during development but also regulators of neuroprotective physiological functions of neurons (68). The LOX family proteins are copper-dependent monoamine oxidases that are mainly involved in the polymerization of collagen and elastin in the extracellular matrix (ECM), hence increase the stability of ECM (69). The expression of LOX family genes is influenced by the IDH1 status of gliomas (70). LOXL1 increases aggressiveness of gliomas by affecting the anti-apoptotic ability of Wnt/β-linked protein signaling (71). Another study found that LOXL1 stabilizes the co-protein BAG2 by blocking K186 ubiquitination, which enables glioma cells to resist apoptosis under non-adherent conditions (72). In contrast, few studies have reported the role of LOXL1 in degenerative neurological diseases. One hypothesis is that LOXL1 protein for aggregates and is actively cleared by autophagy in cells from patients with shedding syndrome (XFG), a cellular defect also found in neurodegenerative diseases such as AD and PD (73).

For GBMs, the mainstay postoperative treatment is the Stupp regimen, i.e. temozolomide concurrent radiotherapy + temozolomide adjuvant chemotherapy. However, the extremely heterogeneous and aggressive nature of GBM results in low survival rate and high recurrence in a large number of patients (74). Similarly, levodopa preparations are the most effective and commonly used drugs for PD, although the disease is incurable and there is no effective drug to delay the progression of the disease. Immunotherapy has emerged as a potential treatment for various diseases, especially for neurological diseases (75). Extensive characterization of the tumor microenvironment (TME) is essential to the identification of reliable prognostic markers and immunotherapy targets in GBM. The high heterogeneity of GBM and the inherent immune evasion mechanism of tumors lead to poor outcomes of GBM patients receiving immunotherapy. In addition, GBM patients have poor prognosis due to the low PD-L1 expression, low tumor mutational load, and depletion of tumor-infiltrating T cells (76, 77).

In the TCGA cohort, several forms of immune infiltration prediction and immunotherapy prediction models were developed. We found that patients in the low-risk group had better prognosis and immunotherapy outcomes. The developed NFRGs risk score model was found to accurately predict the prognosis of patients with GBM, and column line graphs based on this model can help doctors in developing customized targeted treatments. Currently, despite many clinical trials on immunotherapy, there are the efficacy of immunotherapy for GBM is not well understood. Even though it appears to be the most effective method of treating Parkinson’s syndrome, immunotherapy for PD is yet to be clinically applied due to limited evidence. In future, experimental and clinical cohort studies should explore the associated molecular pathways based on the present findings. Such studies will have significant therapeutic value and promote the application of precision medicine in GBM and PD patients.

## Data availability statement

The original contributions presented in the study are included in the article/**Supplementary Material**. Further inquiries can be directed to the corresponding authors.

## Author contributions

SZ and CW conceived the study. SZ, CW, HC, QY, SC, KX, KS, GP, HL and ZX drafted the manuscript. HC, GL, KX and HL performed the literature search and collected the data. SZ, CW, HC, CC and KS analyzed and visualized the data. SZ and CW designed and completed in vitro experiments. HC, GL, PL and ZX helped with the final revision of this manuscript. All authors contributed to the article and approved the submitted version.
