# Supplementary material for: Identification and validation of neurotrophic factor-related gene signatures in glioblastoma and Parkinson’s disease
Source: Front Immunol. 2023 Feb 7;14:1090040. doi: 10.3389/fimmu.2023.1090040 (PMC9941742; doi:10.3389/fimmu.2023.1090040)
Supplement: Supplementary file 1 [file DataSheet_1.pdf]

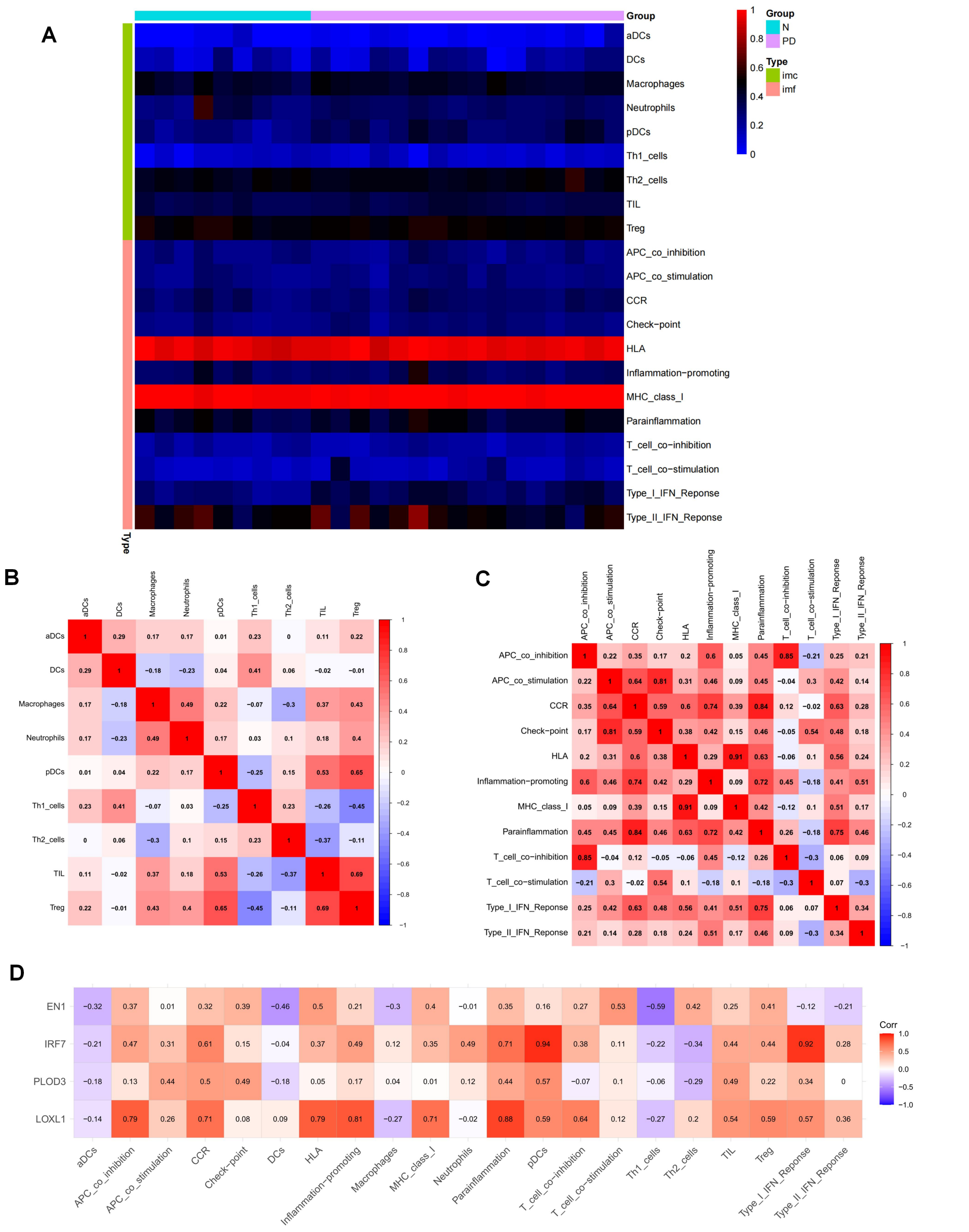

Figure S1. ***Immune cell infiltration analysis.*** ***(A)*** *Immune cell and immunefunction heat mapsfor the PD group and the healthy control group.* ***(B, C)*** *Correlation matrix of immune cells and immune function. Red indicates a positive correlation, blue indicates a negative correlation, and darker color indicates a stronger correlation.* ***(D)*** *Correlation analysis ofthefour characteristic NFRGs and immune cells and immune function.*

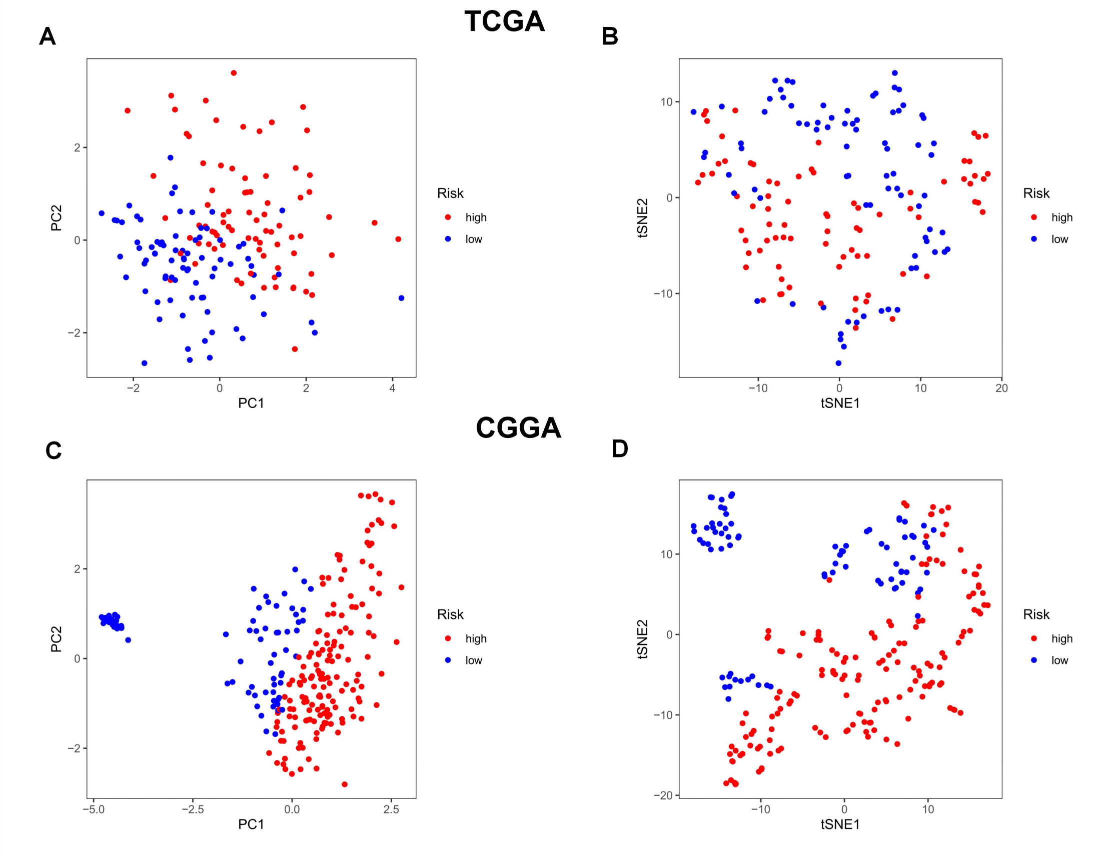

Figure S2. PCA plot and t-SNE analysis in both TCGA and CGGA cohorts. *(A) PCA plot in TCGA cohort. (B) t-SNE analysis in TCGA cohort. (C) PCA plot in CGGA cohort. (D) t-SNE analysis in CGGA cohort.*

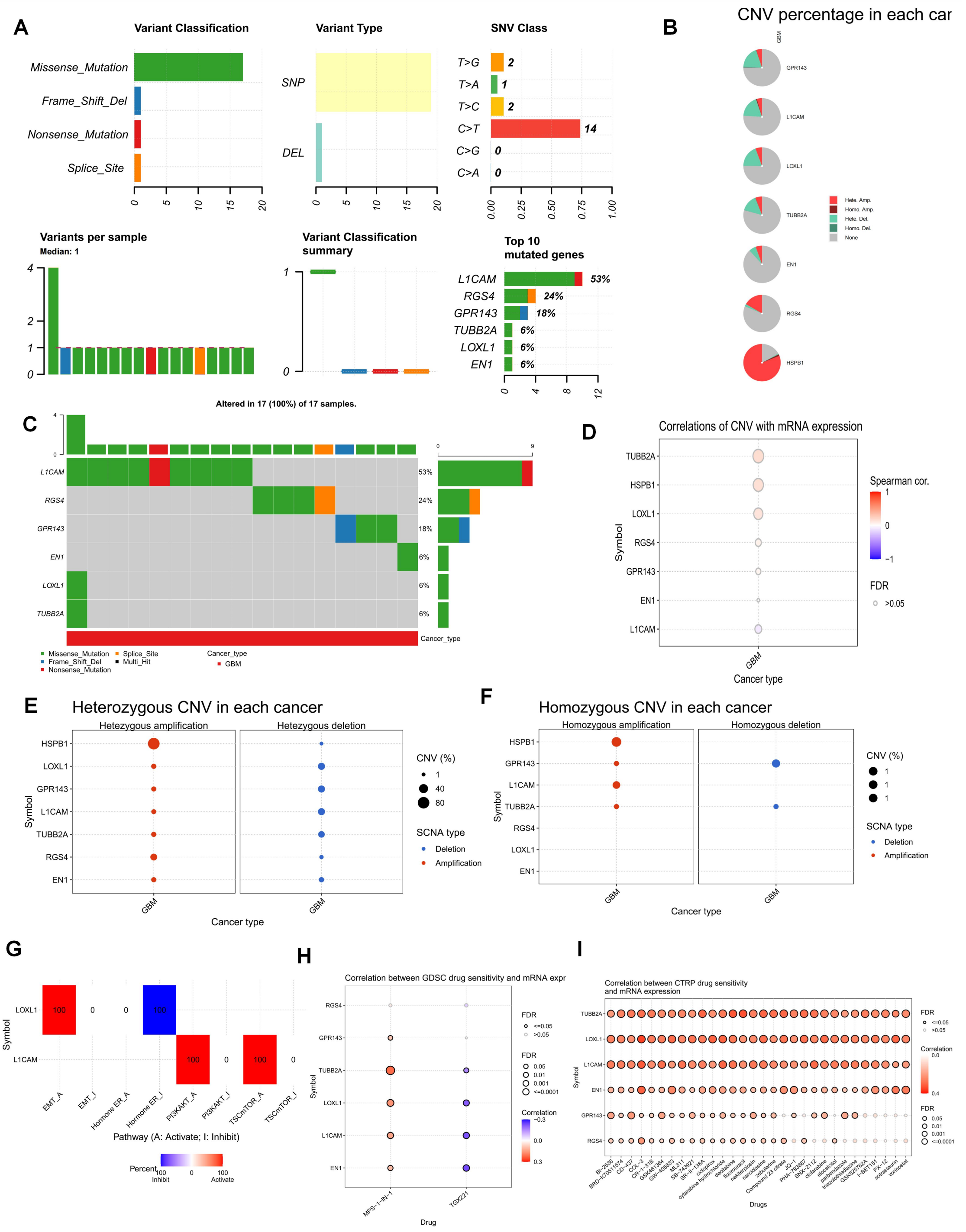

Figure S3. Multi-omics mutation characteristics of NFRGs. *(A, B) Classification of mutations in NFRGs in GBM and their mutation incidence. (C) The proportion of different types of copy number variants in NFRGs. (D) Correlation analysis of copy number variants of NFRGs with expression. (E, F) Distribution of copy number variants amplification and deletion of NFRGs in Homozygous mutation and heterozygous mutations. (G) Analysis of the role of NFRGs' expression activity in the regulation of cancer-related pathways. (H, I) Correlation analysis of NFRGs' expression and sensitivity to chemotherapeutic drugs in CDRP and CDSC cohorts.*

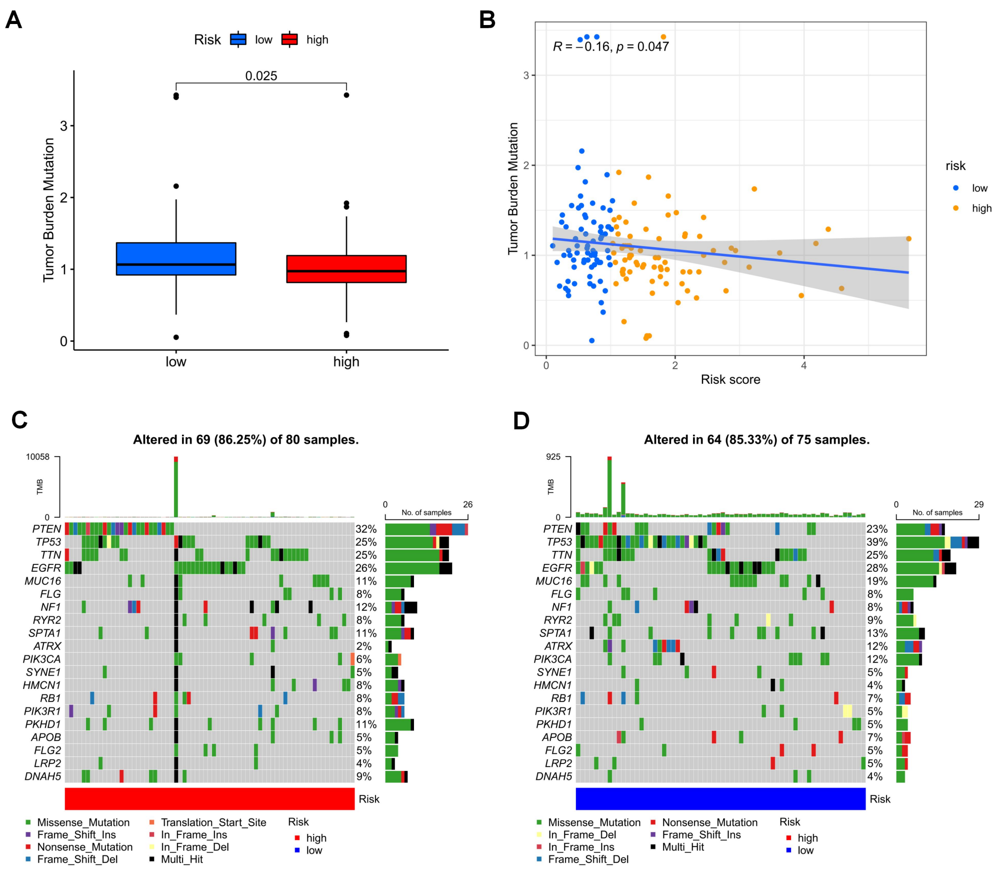

Figure S4*. *Mutation landscapes based on risk score models. *(A) Tumor mutation loads differ between groups with high and low risk scores. (B) Correlation of risk score and TMB. (C,D) summarizing the mutations in high- and low-riskpatients using waterfallplots.*

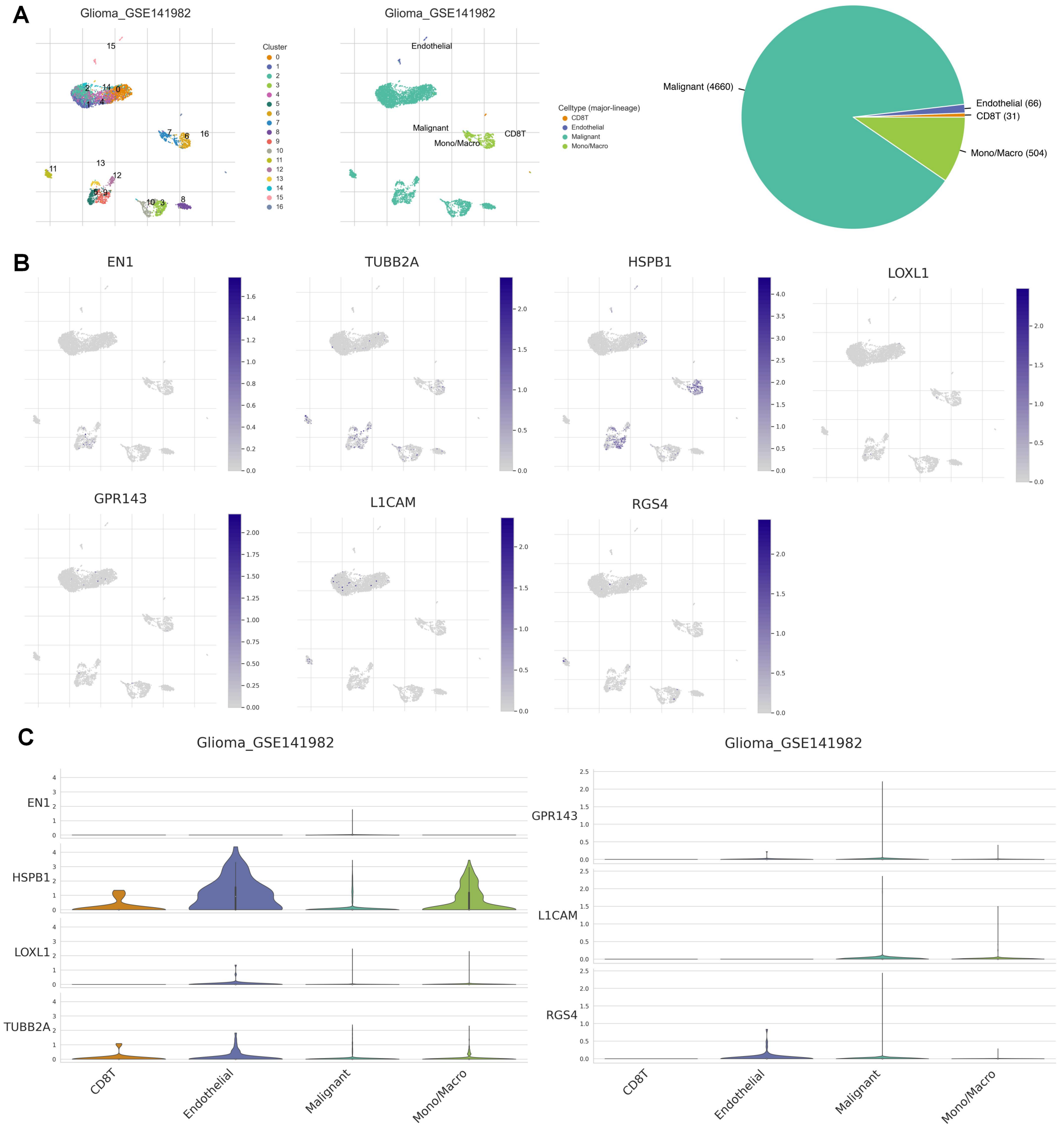

Figure S5. 7-NFRGs in single-cell RNA sequencing. *(A) Annotation of all cell types in GSE141982 and the percentage of each cell type. (B, C) Expression of EN1, HSPB1, LOXL1, TUBB2A, GPR143, L1CAM, and RGS4 in each cell type.*

Table S1.Results of univariate and multivariate Cox analysis in the CGGA cohort

unixoc	id	HR	HR.95L	HR.95H	pvalue
	Gender	1.073123671	0.843961161	1.364511148	0.564745275
	Age	1.007471216	0.998612148	1.016408877	0.098580987
	IDH_status	1.368698278	1.030661992	1.817603629	0.030109844
	MGMTp_methylation	1.101797197	0.870693644	1.394241329	0.419592901
	riskScore	1.247845234	1.077125508	1.445623297	0.003180471

multiCox	id	HR	HR.95L	HR.95H	pvalue
	Gender	1.055861087	0.826688651	1.348564096	0.663266991
	Age	1.00558586	0.996629935	1.014622265	0.222320362
	IDH_status	1.155415696	0.845051012	1.579769045	0.365404338
	MGMTp_methylation	1.048644241	0.822823519	1.336440583	0.701069693
	riskScore	1.199107921	1.022143128	1.406710828	0.025827443
